# How not to write a constitution: lessons from Chile

**DOI:** 10.1007/s11127-023-01046-z

**Published:** 2023-02-22

**Authors:** Guillermo Larrain, Gabriel Negretto, Stefan Voigt

**Affiliations:** 1 Faculty of Economics and Business and LEXEN, University of Chile, Santiago, Chile; 2 Pontifical Catholic University of Chile, Santiago, Chile; 3 University of Hamburg, Hamburg, Germany; 4 CES-Ifo, Munich, Germany

**Keywords:** Democracy, Constitution, Constitutional convention, Independents, Political parties, Constitution-writing rules, Chile

## Abstract

On September 4, 2022, Chilean voters massively turned down a constitutional proposal that responded to widely shared criticisms of the 1980 constitution and emerged from a consensual and participatory process. This result is paradoxical because ex ante, the odds seemed largely in favor of changing the status quo. We argue that three factors, which derived from the interaction between rules and political contingency, explain the outcome: a Convention under the control of party-less independents, the exceptional underrepresentation of the political right, and a highly decentralized and public writing process. We extract some lessons from the failed experience that can be useful for countries seeking to deepen democratization through constitutional change and for a future constitution-making process in Chile.

## Introduction

On September 4, 2022, Chilean voters turned down the draft constitution that had been prepared by an elected convention by 62–38%. This result seems paradoxical. Chilean voters had to decide between the proposed constitution or sticking with the currently valid one created in 1980. Although the existing constitution had served the country relatively well in terms of political stability and overall economic growth in the past, the status quo did not seem acceptable to a large majority of the people at the beginning of the process. Many Chileans had expressed their discontent with the current state of affairs during the violent demonstrations of October 2019 and in the plebiscite of October 2020, where 78% of voters supported replacing Pinochet’s constitution. This created the impression that the win set, i.e., the set of alternatives that a majority would prefer to the status quo, was quite large.

The Convention’s proposal responded to widely shared criticisms of the 1980 constitution by expanding social rights, decentralizing power, strengthening legislative majorities, fostering pluralism and social inclusion, and allowing direct citizen participation in collective decision-making. In addition, the draft emerged from a highly consensual and participatory constitution-making process. However, some reforms in the list and types of rights and in the nature of the state proved to be highly divisive in society and the process was tainted by conflicts and polarized debates among delegates. In the end, although a vast majority of Chileans still preferred a new constitution after rejecting the proposal, they did not consider the draft was as an improvement over the status quo and preferred to wait for a better constitution in the future. Why did Chileans reject the proposal and what lessons does the process provide for countries seeking to deepen democratization through constitutional change?

We argue that three factors, which derived from the interaction between rules and political contingency, explain the outcome: a Convention under the control of party-less independents, the exceptional underrepresentation of the political right, and a highly decentralized and public writing process. In the context of an acute crisis of representation, the rules created for the selection of delegates allowed voting for lists of independents. Instead of overcoming the crisis, these selection rules exacerbated it by facilitating a large proportion of votes for candidates without party leadership and comprehensive reform programs, most of them on the left. Parties on the right, in turn, had an exceptionally bad performance in the election to the Constitutional Convention, which was held under voluntary voting at a particularly critical juncture. This led to an assembly and a constitutional proposal to the left of the median voter of the electorate at large that participated in a ratification referendum held under compulsory voting. Finally, a highly decentralized and public writing process in which committees using simple majority voting were able to put forward radical proposals that had no chance to be approved in the plenary contributed to the image of a conflictive and disorganized assembly in the eyes of the public.

The failed constitution-making process in Chile offers some lessons for constitution writing. The most important is the need to include and possibly strengthen the presence of partisan organizations as central actors in constitution making. They are responsible for forging and sustaining compromises during the writing of a constitution and for informing and mobilizing voters if the final text must be approved by popular vote. The solution to the existence of weak parties is not to abandon parties as the main agents of representation. The second lesson is the need to write a constitution that takes the preferences of all important political groups explicitly into account, even those whose representation is temporarily affected by electoral results. Lastly, even constitutions that seek to strengthen democratic legitimacy must be written in a process that fosters negotiation, centralized coordination over the elaboration of the draft, and a balance between secrecy and publicity.

## The road to the convention

A commission appointed by a military junta headed by General Pinochet wrote the 1980 constitution in a secret, executive-led non-participatory process. The text was approved in a plebiscite which lacked the minimum standards of fairness. After the inauguration of democracy in 1990, the constitution was reformed several times to remove most of its authoritarian features. The most important of these reforms took place in 1989 and 2005. Nevertheless, the constitution maintained central aspects of its original design: centralization of legislative power in the presidency, strong restrictions on the ability of legislative majorities to change policy, weak protection for social rights, and absence of mechanisms of popular participation.

By 2009, however, after the country had experienced almost two decades of impressive economic growth and political stability, growing disaffection with the constitution was apparent. In the 2009 presidential election, all center-left and left-wing candidates advanced the idea of major constitutional reforms or a new constitution. In 2013, a grassroots campaign called “*Marca tu voto*” (Mark your vote) was organized with the aim that in that year’s election for president of the republic, votes marked “AC” for Constituent Assembly would be considered valid as an expression of support for the election of such a body. The initiative failed but the debate reappeared during the presidential election that year.

The right-wing presidential candidate, Evelyn Matthei, mentioned some reforms during the campaign, while the center-left candidate Michelle Bachelet, who eventually won the election, proposed a “new constitution” carried out through a “democratic, institutional and participatory” process. Bachelet managed to organize public consultations on reforms and eventually submitted a draft constitution to Congress by the end of her government. Yet the process was never formally activated due to insufficient support across parties, particularly on the right. For the 2017 presidential election, center-right candidate Sebastián Piñera proposed the introduction of some reforms, such as the popular initiative of law, regional plebiscites, and constitutional recognition of indigenous peoples. But he disdained the idea of a new constitution and after winning the election he not only discarded Bachelet’s proposal but also buried the whole process.

Piñera’s position changed as result of the social mobilizations of 2019. On October 14, 2019, a panel of experts recommended that the cost of a subway ticket should be increased by 30 Chilean pesos, or 4 cents of the dollar. The same day, the first acts of refusal to pay for subway entrance were observed. One week later, this had become a massive practice, particularly among young students. In the immediate aftermath of those events, there was no political message conveyed to the public arena but soon some demands, such as better pensions and improvement to the provision of public health and education, became apparent. Violent acts of protest erupted in many cities and social unrest became the norm. On the 19th, the government declared a state of emergency including the first curfew since the dictatorship.

In the context of a totally disrupted social life, on November 15 party leaders across the ideological spectrum reached the Agreement for Social Peace and a New Constitution (*Acuerdo por la Paz Social y la Nueva Constitución*). This agreement was signed by all parties represented in Congress except the Communist Party and some smaller parties belonging to the left-wing Frente Amplio. The agreement called for a plebiscite to be held in April 2020, although it was later postponed to October because of the COVID-19 pandemic. In this plebiscite people decided on whether to have a new constitution and what type of constitutional assembly would write it. The result was 78% in favor of drafting a new constitution and 80% supporting that all members of the Convention had to be elected (thus excluding members of Congress). In the context of a long-term trend in declining voter turnout, the entry plebiscite represented an improvement, with a turnout slightly above 50%.

The original agreement was complemented by a constitutional amendment in 2019 that established the basic rules of the process. The convention would have 155 members (the same number as the lower chamber of the legislature) elected by a D’Hondt proportional formula in 28 districts. The convention would not be able to alter its mandate and had to respect the republican nature of the Chilean state, judicial decisions, and international treaties. The norms of the new constitution and the rules to approve them would be adopted by a two-thirds majority of the convention. It also established that delegates to the convention could not compete for elected positions for a year after the end of their commission, that the convention should complete its work in 9 months (with a possible extension to 12), and that a popular referendum would be required for the final enactment of the new text. Unlike the initial referendum to authorize replacement of the constitution, which was held under voluntary voting, the ratification referendum required compulsory voting with the objective of preventing the relatively high levels of electoral abstention prevalent in the country.

Further reforms adopted in 2020 sought to emphasize political pluralism, social diversity, and non-partisan representation. These were critical reforms, as we shall later see. One put groups of independent candidates on a similar footing to political parties, by allowing them to compete with their own lists. Another secured gender parity by requiring the inclusion of an equal number of men and women as candidates in each list when the number of candidates in the district was even; if it was uneven, there could be only a difference of one in the number of men and women. Similar rules were applied for the distribution of seats. Finally, 17 out of the 155 seats were reserved for indigenous peoples, also subject to the rule of gender parity. Representatives of indigenous peoples were chosen using a second voting register made of people of self-declared indigenous descent. Voting in this register was voluntary. The 17 reserved seats were created assuming that all indigenous peoples would vote in the second register, something that did not happen, and which led to their overrepresentation.

## The convention: composition and rules

The results of the convention election on May 15 and 16, 2021, did indeed lead to a socially and culturally plural assembly, with an equal proportion of seats for men and women, representation from indigenous groups, and delegates with a wide diversity of educational backgrounds and professions. From the perspective of the individuals that composed it, the Chilean Convention was reasonably endowed with technical capability. For instance, there were 59 lawyers among the 155 members. There were also teachers, engineers, journalists, psychologists, doctors, actors, social workers, entrepreneurs, an economist, a political scientist, a farmer, an accountant, a business administrator, a dentist, an architect, a geographer, and even a chess player. Only nine delegates lacked any form of completed tertiary education. From the point of view of institutional experience, there were six former members of parliament and nine former authorities of the executive branch.^1^

From the point of view of political representation, there were three salient features: the large proportion of non-partisan candidates elected, the high level of party system fragmentation, and the poor performance of parties on the right. While the rules for the election of the convention facilitated the vote for independent lists, these lists (plus one independent outside list) obtained 40% of the total vote and 48 (31%) seats. Yet, the number of non-partisan delegates was much higher: 39 independents ran associated with party lists and the 17 indigenous representatives were also independent of political parties. This means that the total number of independent representatives was equivalent to 104 or 67% of the Convention. Party fragmentation also increased as the most voted party list declined from 38.6% of the vote in the last legislative election (2017) to just 20.6% in the convention election. Finally, the distribution of ideological preferences in the Convention showed a clear bias to the left.

As shown in Table 1, the center-right and right coalition (*Vamos por Chile*) was the most voted list and obtained 37 seats (24%). The leftist coalition (*Lista Apruebo Dignidad*) outperformed the traditional center-left (*Lista del Apruebo*) that had governed Chile from 1990 to 2010; they obtained 28 and 25 seats, respectively. Among the independents and independent lists, which together obtained 48 seats, the vast majority was also located on the center-left or left of the ideological spectrum. The most salient list among them was the *Lista del Pueblo*, a leftist group linked to various social movements involved in the 2019 protests, which obtained 24 seats, followed by a more centrist group, *Independientes no Neutrales*, with 11 seats. While the ideological preferences of indigenous representatives were mixed, the majority had left of center or leftist views. At the time of the convention election, in May 2021, it was unclear whether this ideological balance signaled a lasting shift to the left in the distribution of political preferences among voters. As it turned out, however, the convention election was indeed exceptional and not part of a new trend.

**Table 1:** Constitutional convention initial composition

Representatives	Ideology	Total seats (%)	Independents (%)
Vamos por Chile*	Center-right/right	37 (23.87)	15 (9.68)
Apruebo dignidad*	Left/communist	28 (18.06)	12 (7.74)
Lista del apruebo*	Center-left	25 (16.13)	12 (7.74)
Lista del pueblo**	Left	24 (15.48)	24 (15.48)
Reserved seats	Mixed	17 (10.96)	17 (10.96)
Various independents***	Center-left/left	13 (8.39)	13 (8.39)
Independientes no neutrales**	Center-left	11 (7.10)	11 (7.10)
Total		155 (100)	104 (67)

*Party list; **Independent list; ***Independent lists + one independent outside lists

At the time of the convention election, the performance of parties on the center-right and right was affected by the negative image of Piñera´s presidency, which they supported. Piñera was experiencing very low levels of approval and increasingly high levels of public disapproval since late 2019, first due to the government´s mishandling of social mobilizations and public order and then to the negative economic and social effects of the pandemic.^2^ In addition, the purpose of the convention election was to select delegates who would approve reforms to the existing constitution. Yet the center-right and right parties had been traditionally against changing the 1980 constitution and had no reform proposals to mobilize the electorate. Finally, the convention election, like the initial referendum to authorize the replacement of the constitution, was held under voluntary voting so that those most disaffected with the status quo were the most likely to vote. In total, only 41.5% of the electorate participated.

The confirmation that the convention election was exceptional was revealed in the legislative and presidential elections held in November 2021, six months after the Convention was formed. In the legislative elections, the center-right and right parties obtained 36.6% of the vote and 43.9% of the seats in the Chamber of Deputies, a proportion very similar to the 36.7% of the vote and the 46.4% of deputies they had obtained in the 2017 legislative election.^3^ In the presidential election, the main contender to the center-left candidate Gabriel Boric was the rightist Jose Antonio Kast, who ended up first in the first round but lost in the runoff.

Election rules and election results must be considered in association with the working rules adopted to produce the constitutional proposal. These rules were in part established beforehand, in Articles 130 to 143 of the existing constitution, and in part by the rules of procedure that the Convention itself passed between July and September 2021. The main rule adopted from the beginning of the process, and later incorporated into the constitution and the rules of procedure of the Convention, was the threshold of two-thirds of the votes of the membership in the plenary to pass either individual provisions or the full text of the proposal. So was the requirement that the proposal had to be ratified by a popular referendum to be held under mandatory voting.These mechanisms of popular participation were later complemented by rules of procedure that made possible the presentation of popular initiatives to the Convention and required delegates to hold public forums in their districts to discuss their work during the process.

According to Article 133 of the constitution, the president and vice-president of the Convention had to be elected by absolute majority. Each candidate was elected based on a version of approval voting, and unlike most constitution-making bodies—which tend to allocate authority positions based on the vote and seat share of each party or coalition—the presidency and the vice-presidency were not distributed among candidates from the two most voted political groups. Once the authorities were elected, they proposed that the board be expanded to seven vice-presidents, all of whom were selected using the same electoral method. To secure a high degree of transparency—and supposedly also accountability—the proceedings of the assembly would be transmitted direct.

The work was divided among seven committees organized (simplifying the names) around the following substantive topics: Political System, Constitutional Principles (with two subcommittees), Form of State (with two subcommittees), Fundamental Rights (with four subcommittees), Environment and Economic Model, Systems of Justice, and Systems of Knowledge. Two additional committees specialized in Popular Participation and Rights of Indigenous Peoples. Each elected member of the convention was free to choose the committee(s) to which to belong and, just as in the case of the authorities of the convention, authority positions within committees were not allocated in proportion to the relative political weight of the different groups.

The decision rule in the committees contrasted with that of the plenary. Whereas the latter could only make decisions by two-thirds of its members, the former approved proposals by simple majority, that is, a majority of those present and voting. In part due to the commitment to publicity and openness, no deadlock-breaking device or mechanism of formal political negotiation was devised in the event the committees’ proposals did not reach the two-thirds required in the plenary. The drafting process was highly decentralized. There was no initial draft or set of specific guidelines to work with. Each committee started from scratch and would send separate reports to the plenary, to be voted first overall (votación en general). If the report was rejected, it went back to the committee, which would submit a new revised version to the plenary; if the plenary approved, then it would vote provision by provision (votación en particular). Integration of the different reports took place only at the very end, after each had been approved separately. The plenary never deliberated or voted on a single, unified draft.

## Rules and politics interacting

With the composition, rules, and structure described in the previous section, the Convention started to work within each committee by the end of 2021. Between March and May 2022, the plenary voted on the different reports of each committee, in a complex iteration between plenary and committees. The draft was completed in June and published on July 4. It was a lengthy constitutional proposal (even by Latin American standards) of 388 provisions.^4^

The proposal contained some continuities with the existing constitution, such as the presidential structure of government, but it also included several innovations. Some of them were expected and in line with the context from which the process emerged after the social mobilizations of 2019 and previous criticisms to the 1980 constitution: the creation of new socio-economic rights, greater representation of women and indigenous groups in the legislature, inclusion of mechanisms of popular participation, weakening of presidential powers, and a decentralization scheme. Other changes, however, were more controversial, such as constitutionalization of the right to abortion, the creation of a plurinational state with vaguely different judicial and legal systems for indigenous peoples and other Chileans, potentially weaker protection of property rights in case of expropriation, or the right of unions to strike for any reason they see fit.

**Fig. 1:**
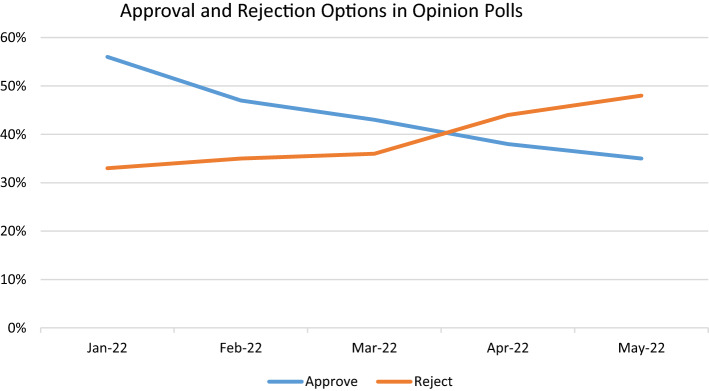
Approval and rejection rates according to opinion polls. Source: Authors, based on Aylwin et al. (2022)

As shown in Fig. 1, the mean level of public support for approval of the new constitution in the future ratification referendum was 56% in January 2022, before the first reports with provisions approved in the committees were known. However, between February and May 2022, when committee reports were submitted to the plenary and the first draft of the constitution was taking shape, support for the new constitution declined from 47 to 35%. Meanwhile, the percentage of those surveyed willing to reject the proposal increased steadily. A similar trend occurred with levels of public trust in the Convention, which went from 51 to 43% between January and May 2022.^5^ In spite of the fact that the proposal completed in June ended up responding to long-standing social demands in Chile, these trends anticipated the defeat of the proposal.

Could delegates have reacted to these polls, by moderating or withdrawing the most controversial proposals? Although there was uncertainty about the results of a ratification referendum held under mandatory voting and thus with an electorate potentially different from the one that elected the Convention, it seems plausible that a majority of delegates never perceived the 1980 constitution as a realistic default outcome. After all, large majorities had been in favor of a new constitution both in the 2020 referendum and in successive polls. In addition, while the Convention was in session, leftist candidate Gabriel Boric was elected president, a result that may have been interpreted as yet another signal the Chileans were in favor of far-reaching change.

We think that the defeat of the proposal was the result of an interaction between ill-designed rules and contingent political events. Ex ante, several key rules adopted for election of delegates and writing of the draft seemed desirable, as they sought to foster social inclusion, consensus, and citizen participation. Yet the way some of these rules interacted with the political forces represented in the Convention produced some unexpected negative results. The rules that facilitated the election of independent convention members created an atomized assembly without organizations and leadership able to forge or sustain coherent agreements throughout the process. The need to secure two-thirds of the vote in the plenary did in the end moderate the most radical proposals produced in committees that made decisions by simple majority. Yet the weak representation of political groups on the right and their exclusion from authority positions in the convention made it unlikely that their preferences were seriously considered in the final draft, leading to their alienation early on in the process. In addition, a writing process that was both highly decentralized and public contributed to the image of a conflicted and disorganized assembly.

### A non-partisan convention

Writing a constitution is an act of cooperative decision-making that involves several mechanisms to aggregate or transform individual preferences into a collective choice (see Elster 1995; Voigt, 1997). The traditional organizations that act as vehicles for the articulation of preferences of constitution makers are political parties. Parties provide positions across different issues and dimensions. Party leaders, in turn, forge compromises and organize voting. As party representatives are likely to be present not only in a special body temporarily responsible for drafting the constitution but also in institutions at the post-constitutional stage that will implement the new constitution over time, they have a stake in both the design and enforcement of the new constitution.^6^ In addition, if the draft constitution must be ratified in a referendum, parties could be central for voter mobilization in support of the draft version.

After democracy was recovered, political parties in Chile enjoyed massive support. According to the World Values Survey, between 1989 and 1993, half of the population had confidence in political parties. This support has suffered steadily since then. When the survey was conducted for the 7th Wave between 2017 and 2022, support had declined to 17.6%.^7^ Several rules for the election of the Convention aggravated this situation. The most important was the rule enabling the vote for lists of independents. Groups of independents lacked organization and consistent reform programs. Most independents were single-issue activists seeking to participate in the Convention as environmentalists, feminists, or traditionalists rather than as agents responsible for negotiating across multiple, complex dimensions. For this reason, groups of independents tended to fragment further in the convention, even when they were initially elected in a single list.

In theory, there are arguments in favor of electing delegates to a constitutional convention on an individual basis. In his treatise on constitutional democracy, Mueller (1996), for example, argues that electing individuals rather than parties is likely to lead to better results as parties would have particular interests *after* the convention which might influence their behavior *during* the convention’s deliberation. In practice, however, electing non-partisan delegates does not guarantee their impartiality and may reinforce a tendency of independents to focus on the single issues they advocated during the electoral campaign. Agreeing on a constitution necessitates many negotiations and compromises which are difficult to attain if delegates only care about isolated, discrete reforms.

Excluding the possibility that delegates to the convention compete for future legislative positions was another rule that reinforced non-coordinated solo behavior. In theory, again, one expectation regarding delegates who will not be legislators later on is that they will assume a long-term view. They are not subject to a re-election constraint implying that they do not need to cater to any short-term preferences voters might have. However, as delegates were not going to be parliamentarians later on, they were essentially playing a non-repeated game. This did impede their willingness to compromise (“you get something now if you do me a favor in the next round” was excluded). On the other hand, knowing that they will not become legislators later on may also imply a preference to bind future legislators as closely as possible. This may be one explanation for the length of the draft, which would have been one of the longest constitutions in the world.

### Underrepresentation and exclusion of the right

As reported above, the political right had a mere 24% of the delegates to the Convention, a share below the representation they had in legislative elections before and after the convention election. The traditional center-left that had governed the country after the democratic transition, also experienced an electoral decline in the convention election, but it was less exceptional. They had been losing support since 2017 against the rise of new parties on the center-left and left and did not recover as much in the November 2021 elections. Nevertheless, the weak electoral performance of established parties on both the right and the left in the convention election is consistent with the fact that in this election held under voluntary voting those who participated were likely to be the most motivated to change the status quo.

 The most immediate consequence of the weak representation of the right was that it was unable to block any reform and thus induce negotiations because it did not reach by itself the required 33% + 1 votes. Had the right achieved that threshold the constitutional proposal would have been less radical in content. In addition, divisions within parties on the right and potential allies in the traditional center-left limited its capacity to build a coalition such that it would reach the needed threshold to veto proposals. The alternatives for the right were restricted to negotiating with a portion of moderates of the traditional center-left gathered in the *Lista del Apruebo* and eventually some independents. However, in order to agree on something with them, the most moderate within the right-wing coalition would have lost the support of their far-right colleagues. Indeed, the right was divided in two subgroups, one that showed interest in starting negotiations with the center left, and the other with no intention to bargain at all.^8^ On the other hand, the most important group within the traditional center-left, the Socialist Party, decided early on to negotiate reforms with the left parties in the list *Apruebo Dignidad* because together they were in a better position to win allies to approve reforms by two-thirds in the plenary.

The institutionally weak representation of the right was exacerbated by the absence of a more consensual approach among the leftist delegates. Although the rightist list *Vamos por Chile* did not reach the one-third necessary to block proposals, it was the single largest group in a very fragmented assembly. A more political approach should have led to the inclusion of the preferences of members of this coalition even when there were sufficient votes to override their objections. The governing body of the Convention and authority positions in the committees, for instance, were elected without considering voting power and seat shares, thus leading to underrepresentation of the right in them.

The exclusion of the right had more negative consequences in light of the fact that as the legislative and presidential elections at the end of 2021 showed, parties on the center right and right, despite their poor performance in the convention election, retained their traditional levels of support in the country. Their exclusion from basic negotiations and compromises thus led to an early defensive position of these parties, which started to work for the rejection of the proposal in the ratification referendum well before the Convention had completed its draft.

### A decentralized and public writing process

The two-thirds voting rule to pass any constitutional provision was adopted to secure consensus. This is consistent with the idea that constitutions are the most important formal component of social contracts. Ideally, all citizens who would be subject to a new constitution should consent to it. Unanimous agreement to a constitutional draft is, however, extremely unlikely.^9^ If every delegate has veto power, the temptation to misuse that veto power strategically is very large, which is why supermajorities for the passing of draft constitutions have been suggested (Wicksell, 1896, e.g., proposes a 5/6 majority). Although supermajority rule is not frequently required, it has been used in several recent constitution-making processes, such as those of South Africa, Tunisia, and Nepal (see Negretto, 2017).^10^ In the Chilean case, different voting and approval rules were used. For the final plebiscite, a 50% of valid votes was sufficient. If one thinks of the constitution as a social contract binding all citizens, making the draft pass if a simple majority of citizens approves it appears questionable (Michel & Cofone, 2017) yet it has become somewhat of a convention and is part of the Chilean constitution-making tradition.

Within the constitutional convention proper, two different voting rules were used: in the plenary, proposals needed the support of 2/3 of its members, whereas in the committees created by the convention, simple majority rule was applied. These two different voting rules were inconsistent. There is reason to assume that the various committees were staffed in large part by those who had a rather narrow interest in the topic of their respective committee. The voting rule of the committees was simple majority, so a slim majority was sufficient to get far-reaching proposals accepted. This provided incentives to advance radical proposals to set the agenda for discussions in the plenary, even knowing that those proposals were unlikely to ever be approved there.

The proposal to eliminate the Senate by the committee on the political system provides an appropriate example. When this committee approved the creation of a unicameral legislature by 13 to 12 votes in mid-February 2022, it was apparent that such a proposal would not be approved in the plenary.^11^ Moreover, the first report submitted to the plenary had already abandoned the unicameral legislature and included instead a very weak, almost symbolic second chamber representative of the regions. This provision, along with many others, was rejected by the plenary and sent back to the committee for further negotiations. After subsequent iterations of voting, a stronger second chamber was eventually included in the draft constitution. But the initial unicameral proposal polarized the debate from the very beginning and set the agenda for discussion in future negotiations. The frequent rejection in the plenary of provisions approved in the committees also created an image of disorder and conflict in the eyes of the public, aggravated by the fact that integration of the different committee reports into a single draft did not take place until the very end of the process.

An extreme commitment to publicity exposed the deficiencies of this process. Given that the purpose of a constitution is to facilitate the coordination of all citizens of a country, it seems almost self-evident that the process by which such a constitution is generated should be open to the public. This presumption is also in line with many arguments regarding high quality governance, which would be easier to achieve if there is a high degree of transparency. It could further be argued that a high degree of publicness was desirable because if the public was aware of the arguments exchanged at the assembly, non-members could make themselves heard and participate in the deliberations at least indirectly.

However, if all positions ever taken by all members of the constitutional assembly become public knowledge, this makes it more difficult for assembly members to change their mind. In other words, achieving compromise and consensus on a draft could become more difficult if deliberations are public. Elster (1999) claims that publicity explains some of the failures of the post-1789 French constitutional assembly.

It seems that the combination of individually elected members and live-streamed sessions created incentives for at least some of the convention’s delegates to raise public attention by almost any means. For instance, delegates appeared costumed as Pikachu (one of the characters of the Japanese Pokémon series) and as a dinosaur; a woman who had been treated with breast cancer addressed the convention topless; a singer-songwriter performed a modified version of the Chilean anthem to the delegates and so on. As a consequence, the image of the assembly as being earnestly concerned with the future of Chile was seriously affected.

A Convention without party leadership and organization, a proposal that reflected the preferences of a majority of delegates with leftist views elected in a special election held under voluntary voting, and a drafting process that conveyed an image of conflict and disorder, were all factors contributing to the defeat of the proposal in the ratification referendum of September 4. In this referendum, where voting was compulsory, participation reached 85.86%, a whopping increase compared to the turnout of the convention election. Clearly, in spite of the fact that it entailed a binary choice between the proposal and the existing constitution, the preferences of the median voter in the ratification referendum were closer to those of the median voter in the electorate at large than in the convention election.

Yet, it would be misleading to think that a majority of voters in the ratification referendum opted to maintain the 1980 constitution. Although formally, this was the choice and the result of the rejection, it turns out that Chilean voters perceived a third option. Polls taken after the defeat of the proposed constitution showed that 79% were still in favor of changing the constitution.^12^ This situation is markedly different from a setting in which a country has no valid constitution or one in which there is a shared understanding that the existing constitution is clearly dysfunctional. In these cases, the ex-ante likelihood of a proposal being accepted is very high. But it is also different from a setting in which voters opt between a proposal of dubious quality and an existing functional constitution.^13^ Chileans still want another constitution but not just any other one. For a majority of the voters, the far-reaching changes of the proposal were unpersuasive and generated deep doubts about their implementation. The third option was to be neither in favor of keeping the 1980 constitution nor of the draft constitution but in favor of yet another constitutional assembly.

## Lessons from the Chilean case

What lessons for constitution-making can be drawn from the failed Chilean experience? This assessment is crucial because the Chilean process was initially regarded as exemplary in a region with a penchant for frequent constitutional change but few desirable models of democratic constitution-making. A reflection on the event is also needed because despite the rejection of the proposal, the constitution-making process is far from over in Chile and a new draft must eventually be produced to replace the 1980 constitution. Our conclusions in this regard follow our analysis of the Chilean process described above.

1. Parties as representing people with similar preferences are key in constitution making because they generally have programs covering multiple policy dimensions. This is important for being able to negotiate and reach compromises. Parties are also repeat players who are expected to be represented in post-convention parliaments. For this reason, during the deliberations of constitutional assemblies, party representatives are likely to have the consequences of their decisions for future legislators in mind, which increases the probability that the draft constitution is designed as a workable set of rules.
2. Procedural rules should guarantee that important minority positions cannot simply be outvoted. Voting on the constitution by two-thirds of the Convention was a reasonable rule in Chile. Yet due to its weak electoral performance, the center-right and right list did not command veto power despite being the single largest group. This situation, however, could have been improved by allocating authority positions within the Convention and the committees according to the relative political weight of each group. This would have given representatives on the right the incentives and opportunity to present their proposals, shape the agenda for discussion, and eventually seek agreements. Yet an assembly biased to the left was unwilling to provide this group with such an influence.^14^
3. Although transparency of the deliberations of a constitutional convention seems intuitively desirable, there are important arguments that relativizes it. Compromises imply that delegates to the convention need to agree on outcomes that are not their most preferred ones. If their positions are widely known among the public, agreeing to a compromise might be equivalent to losing face.
4. Combining delegates elected as independents advocating single issues with complete transparency of the meetings seems particularly toxic as this can be misused for personal promotion. It should be prevented.
5. Committees were formed by voluntary adherence. This induced self-selection in which activists played a critical role in committees where they were the least able to bargain, because activists tend to have unidimensional agendas. This made it possible for too many maximalist proposals to be flagged, and the low voting threshold at the committee level allowed some of them to reach even the plenary level. All this harmed the Convention’s reputation.
6. A coordination committee that mediates between conflicting proposals originating from different committees and has an eye on the cohesiveness of the entire draft can prevent incoherent and radical committee proposals from being rejected by the plenary. In Chile such a committee started too late and did not play such a role.
7. Having both an entry and an exit referendum is quite unusual and demanding for the electorate. But given this choice, a lesson from the Chilean experience is that the voting rules of both referendums should be the same. With an initial plebiscite and convention election with voluntary voting and a final ratification referendum with mandatory voting, two different types of electorates participated in the process. This created a potential incongruence between the preferences of voters and constitution makers at the beginning and at the end of the process.

A final reflection on the constitution-making body is in order. Convening a special convention to write a constitution seems inevitable when legislatures have lost public trust, as was the case in Chile. A convention, however, is a fleeting institution in charge of designing the basic rules of the state and the political regime without taking any responsibility for their future implementation. It is not by chance that most democratic constitutions in the world are written by ordinary legislatures with a special electoral mandate for that purpose rather than by independent conventions. Even if the legislature is discredited, as it certainly was in Chile, it should have a larger role in the drafting of a new constitution.

Like most political processes, constitution making cannot be entirely designed. The actual impact of the rules to elect a constituent assembly depends not only on the electoral formula but also on factors that are to some extent exogenous to it, such as the number of parties competing in the election, the number of voters who decide to participate, and the resources that each political group has been able to mobilize in favor of its proposals. These factors, in turn, determine who is elected and how decision-making rules in the assembly work in practice. Due to their traditional opposition to a new constitution and lack of proposals of reform, center-right and right parties in Chile did not win sufficient seats and thus could be marginalized in most decisions despite a relatively high threshold to pass the constitution.

These exogenous factors, however, interacted with some rules that discouraged partisan representation, political compromise, and organized work and which seriously affected the public image of the convention. The rejection of the draft in September 2022 has left Chile at a crossroads because the 1980 constitution has already lost social legitimacy. As of the time of this writing, a multi-party agreement has called for a second convention, which is significantly different from the first one. It will be a reduced body of 50 elected members whose task is to approve a draft proposed by a commission of 24 experts appointed by Congress. Independents can only compete in party lists and the expert commission will have agenda-setting powers over the convention. It seems that some lessons from the previous failure have been learned, thus increasing the chances of success of the future process.

## Footnotes

1 On the professions of delegates, see https://www.biobiochile.cl/especial/una-constitucion-para-chile/noticias/2021/05/20/las-profesiones-y-ocupaciones-de-los-155-constituyentes-varios-abogados-actores-y-una-ajedrecista.shtml.

2 By April 2021, a month before the convention election, the approval of the president was 12.4% and his disapproval 79.1%. See Pulso Ciudadano, in https://chile.activasite.com/wp-content/uploads/2021/04/Pulso_Ciudadano_Abril_Q1_web.pdf

3 The main difference in the share of votes and seats of rightist parties in the 2017 and 2021 legislative elections is that in the latter, a more radical alternative to the traditional right emerged, the Frente Social Cristiano (Christian Social Front).

4 Brazil’s 1988 constitution, one of the longest in Latin America, has 250 articles.

5 Authors’calculations based on mean support for approval of the draft in September 2022 in two polls, CADEM and Pulso Ciudadano.

6 Brennan and Kliemt (1994) argue that political parties are likely to have longer time horizons than individual politicians.

7 World Values Survey, Wave 1, question Q72 adding answers labelled “quite a lot” plus “a great deal.”.

8 One clear example of this happened six weeks after the Convention started. The pro-negotiation group (15) signed a letter stating that the right had an historical debt with the indigenous peoples. However, the other 22 members of the coalition were not willing to sign. See https://www.latercera.com/politica/noticia/convencionales-de-chv-envian-carta-abierta-a-representantes-de-pueblos-originarios-y-hacen-autocritica-chile-tiene-una-deuda-historica-y-nos-comprometemos-a-contribuir-a-su-solucion/

9 In the Chilean Convention there was only 1 out of 399 proposals that reached unanimity.

10 Only 33 of 124 constitution-making bodies created between 1900 and 2015 required a qualified majority to pass the constitution. See Negretto (2017, 2020).

11 See https://www.chileconvencion.cl/news_cconstitucional/sistema-politico-aprueba-estado-plurinacional-regimen-presidencial-atenuado-y-congreso-unicameral/

12 As reported, e.g., here: https://www.elmostrador.cl/noticias/pais/2022/10/17/encuesta-criteria-mayoria-quiere-cambiar-la-constitucion-aunque-prefiere-composicion-mixta-para-escribirla/

13 A reviewer compared the setting in Chile with that of the U.S. in 1787 and suggested that the proposal might have been defeated because despite all its flaws, the 1980 constitution was a clearly functional constitution. As the Articles of Confederation were really dysfunctional, the win set was huge and the likelihood of the U.S. Constitutional Convention producing a draft that was preferred by a majority was correspondingly high. However, as we argue in the text, voters in Chile rejected the proposal without being in favor of keeping the 1980 constitution for the future. See Dougherty (2000) for an in-depth analysis of the problems of the Articles of Confederation and Munger (2015) for a precise description of the concept of the win set.

14 Lesson 1 combined with lesson 2 is in line with cross-country empirical evidence. Negretto and Sanchez-Talanquer (2021) show that draft constitutions that are written by well-established political groups (such as parties) and need the consent of a plurality of such groups are the only factor robustly correlated with higher levels of liberal democracy after the enactment of the new constitution.
